# Artificial-Intelligence-Driven Electromyography Adaptation for Elderly Assistance at Physiological, Functional, and Behavioral Levels

**DOI:** 10.34133/cbsystems.0638

**Published:** 2026-07-15

**Authors:** Jiaqi Xue, Ziqi Li, Xiaoyang Zou, Zijia Qu, Shengjie Yang, Colin Pak Yu Chan, Yanchen Liu, Zhou Zhao, Jing Zhang, Clio Yuen Man Cheng, Haiyang Wang, Kehan Zou, Yafei Zhao, Vivian Weiqun Lou, Ning Xi, King Wai Chiu Lai

**Affiliations:** ^1^ Department of Biomedical Engineering, City University of Hong Kong, Hong Kong 999077, China.; ^2^ Department of Social Work & Social Administration, The University of Hong Kong, Hong Kong 999077, China.; ^3^ Sau Po Centre on Ageing, The University of Hong Kong, Hong Kong 999077, China.; ^4^ Department of Industrial and Manufacturing System Engineering, The University of Hong Kong, Hong Kong 999077, China.

## Abstract

Older adults frequently face difficulties in activities of daily living (ADLs) due to age-related declines in strength, coordination, and perception. Myoelectric control provides an intuitive human–robot interface by translating muscle activity into assistive commands. However, its practical application is still challenged by signal annotation, multijoint coordination, and cross-task generalization. This study proposes a 3-level intelligent framework for multijoint upper-limb assistance based on electromyography (EMG) to support the daily living activities of older adults. At the physiological level, situation-aware labeling protocols matched to different EMG conditions are proposed to reduce annotation ambiguity and improve robustness to signal changes. At the functional level, focusing on elemental joint activities, a deep backbone model is designed to infer both single-joint movements and coordinated multijoint patterns with an accuracy of 95.34%. At the behavioral level, the model is further distilled to support complex ADL tasks with human–robot interactions while continually incorporating new knowledge without catastrophic forgetting. The framework is implemented in real time on an EMG-controlled multijoint robotic system, providing smooth and coordinated assistance in daily activities. Overall, the proposed framework provides a systematic solution for EMG-based multijoint coordination, encompassing the entire pathway from physiological signal processing to functional intent decoding and behavioral adaptation during daily activities. It offers a technical approach to coordinated upper-limb assistance and lays a broader foundation for the design of practical and adaptive assistive systems, contributing to improved autonomy for older adults and supporting the broader societal goal of healthy aging.

## Introduction

**Fig. 1.**
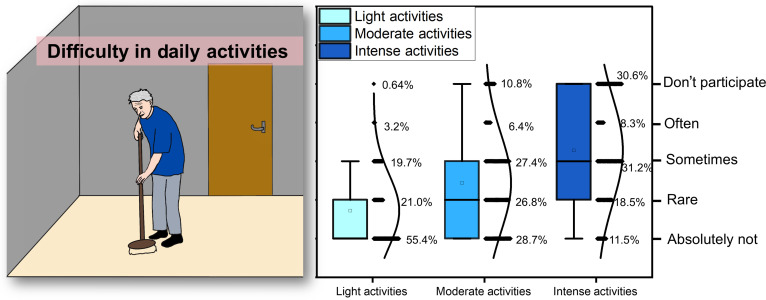
Statistical results of a questionnaire survey in older adults. In total, 157 subjects (67 to 87 years old) were investigated. The left box shows the results of difficulty in light activities. The middle box summarizes the answers concerning difficulty in moderate activities. The right box represents the statistical results of difficulty in intense activities.

Activities of daily living (ADLs), such as doing laundry, getting dressed, and using utensils, are typical and essential for most individuals in their everyday home lives. However, these activities can present great challenges for some groups for independent living. For example, a grandma with muscle atrophy may find it difficult to pick up a pot of soup by herself. Indeed, decreased strength, working memory decline, and hearing and vision loss may occur as individuals age [1–3]. Older adults are more likely to face daily frustrations in their ADLs [4–7]. A survey questionnaire of difficulty (light, moderate, and intense activities) in ADLs was conducted with older adults (157 subjects in total aged between 67 and 87 years) in Hong Kong, as shown in Fig. 1. Three questions are as follows: “Do you have difficulty performing light physical activities (walking slowly, ironing clothes, cleaning, grooming, self-care, watering plants, etc.)”, “Do you have difficulty performing moderate physical activities (brisk walking, window cleaning, vacuuming, car washing, weeding, mopping, Qigong, Tai Chi, etc.)”, and “Do you have difficulty performing intense physical activities (running, climbing, lifting heavy objects, moving furniture, digging holes in the garden, etc.)”. The results indicate that with increasing difficulty, older adults are more likely to frequently feel that it is difficult to finish their daily activities by themselves. More than 8% of people find it challenging to complete intense activities, and more than 30% do not participate in such activities. In general, performing daily activities, especially intense activities, is not easy for older adults. Accordingly, they need additional assistance to support independent and convenient life.

Considering the demands of older adults, scholars have attempted to design assistive devices and systems with advanced technologies. For example, electric wheelchairs are created to help people with mobility issues [8,9]. Prostheses are used to help people who have lost their limbs regain their ability to operate [10–12]. Soft exoskeletons have been proposed to compensate for missing muscle strength [13–15]. With the development of assistive technology, the means to connect human and assistive devices have been a research focus. Electromyography (EMG) signals reveal electrical activities generated by muscles [16,17], which can provide precise information about nerve conduction and muscle function. The generation of EMG usually occurs earlier than muscle movement does, showing the superiority of intent capture in assistive robot control. Therefore, this type of myoelectric signal facilitates intuitive and natural actuation, as the human user’s muscle contractions are directly converted into corresponding device commands. For example, exerting force can be predicted on the basis of EMG signals in human–robot interactions [18,19]. The effectiveness of robot-assisted therapy guided by EMG signals in enhancing limb function has been demonstrated [20,21]. The integration with EMGs in assistive systems has made the system intelligent and user-friendly. However, the realization of such a design generally focuses on the theoretical field, and practical real-time applications still face considerable challenges [22,23].

First, one critical challenge in EMG-based control lies in establishing a reliable mapping between myoelectric signals and motor intent, which largely depends on accurate data labeling. Existing research typically relies on external devices, such as motion capture systems, or manual annotation strategies to obtain ground-truth labels [24–26]. While these methods facilitate data acquisition, they also increase hardware requirements and the workload of annotation [27]. Furthermore, kinematic annotation strategies based on external observations may not fully reflect the underlying neuromuscular characteristics of muscle coordination. In particular, variations in factors such as arm position have been shown to substantially affect EMG signal characteristics and decoding performance [28], which may lead to inconsistencies between externally defined labels and the underlying physiological signals, thereby affecting the consistency and interpretability of the learned representations.

Second, most existing EMG-based activity recognition methods focus on single-joint or independently modeled joint movements [29–31]. While these approaches simplify the modeling process, they fail to capture the coordinated nature of human motion, where multiple joints interact to perform complex tasks. Existing studies have attempted to extend EMG-based recognition to more complex scenarios, including multi-degree-of-freedom prosthetic control and simultaneous estimation of multiple movement variables [32,33]. However, these approaches are typically designed as separate estimations of multiple joint variables rather than true joint coordination, and they rarely reflect the coordinated movement patterns commonly involved in daily activities. As a result, the potential complementary information across joints is underutilized, which limits system performance and robustness.

Furthermore, handling new tasks remains a critical challenge in myoelectric information analysis. Because EMG signals vary across different activities and application scenarios, models trained on predefined tasks often struggle to generalize to unseen conditions [34,35]. Existing methods typically rely on large-scale training datasets designed to cover a variety of scenarios [27,36] or require collecting large amounts of new data and retraining the model to adapt to new tasks when encountering new scenarios [37,38]. While effective in controlled settings, these strategies are data intensive and limit the scalability of EMG-based systems in real-world applications. Furthermore, adapting to new tasks inevitably interferes with previously learned knowledge. In existing methods, this interference is often pronounced, leading to substantial performance degradation and inconsistent behavior across different tasks [39]. These limitations highlight the need for a more efficient mechanism that can adapt to new tasks with minimal additional data while retaining previously acquired knowledge.

**Fig. 2.**
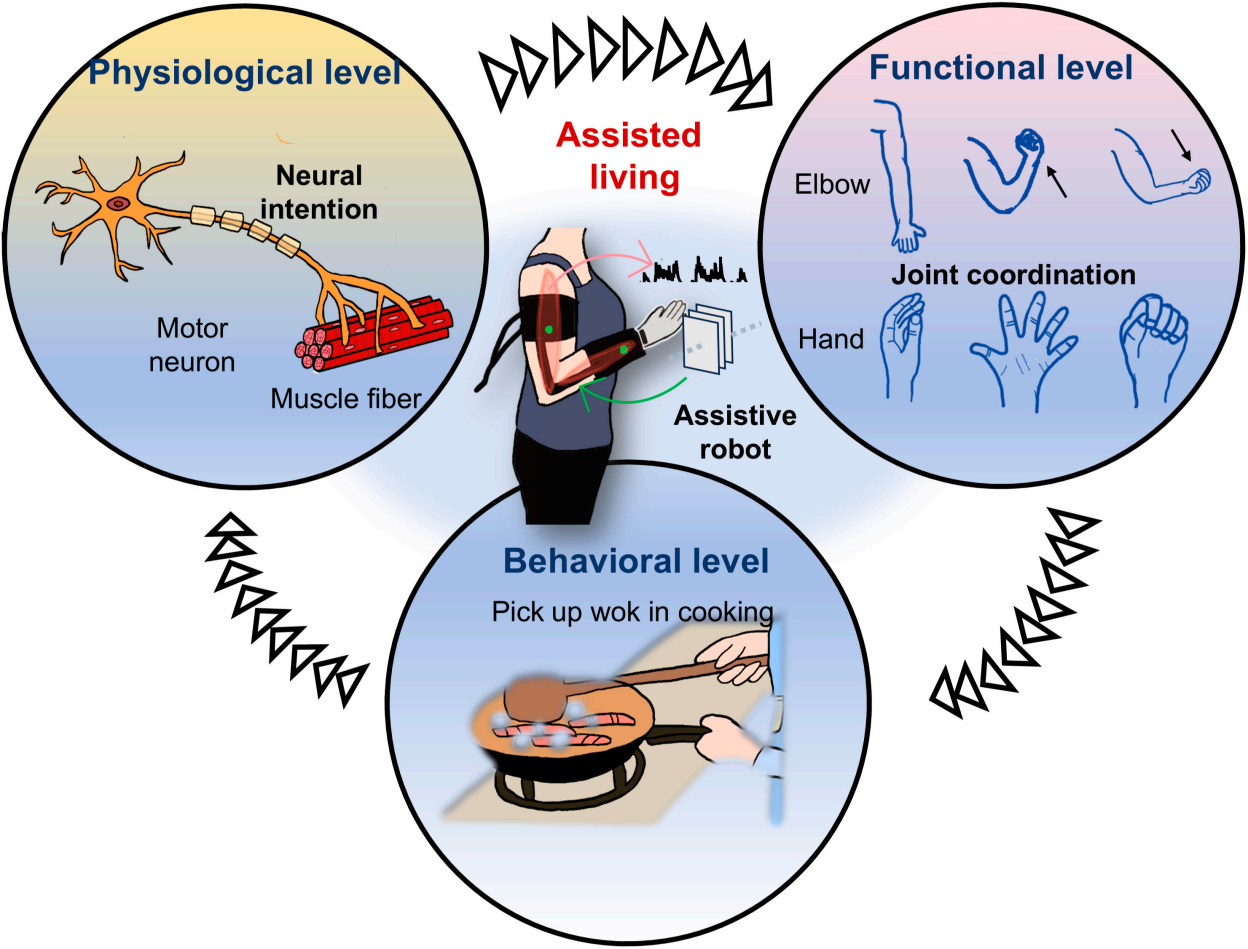
Intent-oriented assisted living at the physiological, functional, and behavioral levels. We propose gradually deepening and designing our assistive system from these 3 levels to meet user needs, specifically from myoelectric information to functional joint intention prediction and, finally, to behavioral applications.

Overall, existing studies tend to address these challenges in isolation, focusing on either labeling strategies, joint modeling, or task adaptation. However, the lack of a unified perspective limits their effectiveness in real-world EMG-based control scenarios. To overcome these limitations, it is necessary to consider the problem across multiple levels. This motivates the proposed following multi-level framework, which systematically addresses these challenges in an integrated manner. Herein, we design a real-time coordinated hand–elbow myoelectric control system that considers a novel artificial intelligence (AI)-driven development approach, as shown in Fig. 2. By introducing model distillation, this system incorporates scenario-specific knowledge while retaining previously learned functional capabilities, thereby supporting flexible adaptation to diverse ADL scenarios. Overall, the system is constructed and implemented across 3 interconnected levels: the physiological level, functional level, and behavioral level.

• At the physiological level, we propose novel labeling strategies based on intrinsic electromyographic signal characteristics and task-specific constraints, aiming to define labels that are more consistent with underlying neuromuscular activity. Unlike traditional methods that rely on external observational kinematics, the proposed strategy better captures the physiological patterns of muscle coordination, thereby improving consistency.
• At the functional level, we aim to capture the coordination of joint movements by identifying basic joint functions from neural information. To this end, we design a compact deep backbone model for efficient intent prediction, enabling the simultaneous learning of multijoint movement patterns. By modeling joint coordination rather than independent joint variables, the proposed method better utilizes complementary information between joints, resulting in a substantial performance improvement compared to a single-task setting.
• At the behavioral level, we leverage knowledge distillation to address the challenge of adapting to new tasks. Considering that complex daily activities can be broken down into basic sets of movements, we designed a distillation-based framework to enhance the adaptability of electromyographic representations. Specifically, we employ self-distillation (SD), inheriting prior knowledge while simultaneously learning new task-specific knowledge. This strategy effectively adapts to new tasks with minimal additional data while minimizing interference with previously learned skills.
  Overall, the proposed 3-level framework can effectively solve key challenges in EMG-based modeling, improving the consistency, coordination, and adaptability of neural representations. These advancements lay a more reliable foundation for deploying intelligent assistive systems in real-world scenarios. In the future, with the integration of assistive robotics technology, such systems are expected to help older adults perform daily activities more autonomously.

## Materials and Methods

### Multi-level systematic framework

**Fig. 3.**
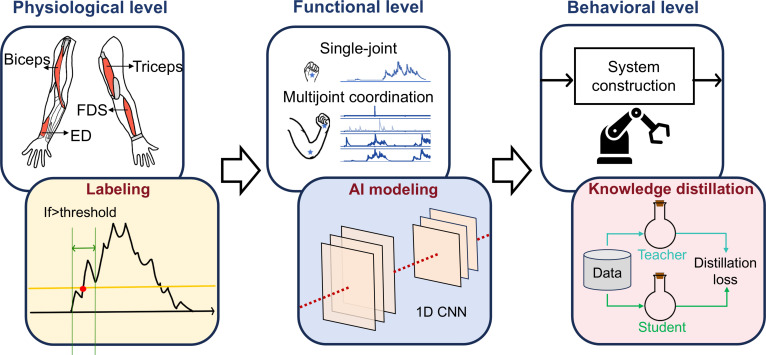
Specific requirements and solutions at 3 levels. First, at the physiological level, electromyography (EMG) signals are collected and labeled on the basis of muscle activity. Next, at the functional level, an artificial intelligence (AI) model is constructed for intent prediction from single-joint movement to multijoint coordination. Finally, at the behavioral level, real-time complex human motions are realized on the basis of the constructed system, in which knowledge distillation is used for learning new tasks.

In this work, an AI-driven myoelectric assistive approach was designed at the physiological, functional, and behavioral levels. In this process, effective methods have been proposed to solve practical problems at each level, as shown in Fig. 3.

At the physiological level, 4 muscles are selected on the basis of muscle activity: the triceps, biceps, extensor digitorum (ED), and flexor digitorum superficialis (FDS). With collected myoelectric signals, the movements of both the hand and elbow are labeled on the basis of muscle activities and interactions. Novel labeling approaches have been designed to address different signal characteristics, reduce manual work in signal processing, and achieve accurate information extraction in model training. Next, at the functional level, the processed myoelectric signals are translated into joint movements from AI model training. For each joint, different joint states are analyzed: open, close, and relaxed and flexed, extended, and relaxed. As the coordination of the hand and elbow plays a necessary role in daily activities, we considered expanding our study from separate single-joint prediction to simultaneous multijoint prediction. Therefore, we design a deep learning model to adapt to joint coordination. Finally, at the behavioral level, we constructed our real-time myoelectric control system by advancing the AI model experience in functional activity to complex human motion. In addition to fundamental joint prediction, we incrementally realized various daily activities, such as picking up and moving while cooking. The knowledge distillation method has been used for the model not only to learn new knowledge in different complex actions but also to retain the old knowledge about joint functions.

### Physiological level: EMG acquisition, processing, and labeling

In this work, we collected a dataset from 10 voluntarily recruited participants, namely, 4 young adults and 6 older adults. Their demographic and clinical/functional characteristics are summarized in Table 1, where the older participants reported more activity-related difficulty.

**Table 1.** Demographic and functional characteristics of participants by age group. Two older participants were unable to perform the handgrip strength test.

Variable	Younger participants (*n* = 4)	Older participants (*n* = 6)
Age	28–32	64–75
Sex (male/female)	3/1	3/3
BMI/(kg·m^−2^)	20.44–22.57	20.92–27.49
Handgrip strength/kg	18.0–50.1	10–31.1 (*n* = 4 tested)
Difficulty in carrying a 5-kg object	0	3/6
Difficulty with light physical activity	1/4	0
Difficulty with moderate physical activity	1/4	4/6
Difficulty with intense physical activity	3/4	5/6

BMI, body mass index

The data acquisition procedure is described as follows: Before the EMG data collection experiment, the skin surface was cleaned with alcohol. Two EMG electrodes were then attached to each muscle, and another electrode was attached to the nerve-free area. The EMG signals were collected from upper-limb muscles, where the triceps and biceps are related to elbow movements and the ED and FDS correspond to hand movements. The protocol of data collection can be divided into 2 parts. In the first part, the participant was asked to perform hand opening with elbow flexion and extension 5 times in a trial, and a total of 5 trials with 1-min relaxing time between trials were performed. In the second part, hand closing with elbow flexion and extension were performed in the same pattern. In this study, elbow flexion and extension refer to the dynamic bending and straightening of the elbow joint during task execution, rather than a predefined static joint position. To ensure the safety of elderly participants, a uniform maximum flexion endpoint was not required. Instead, flexion was considered sufficient when the elbow reached a flexion angle of approximately 120° or greater from the fully extended position, where the fully extended elbow was defined as 0° of flexion. This angle denotes the flexion excursion from full extension, not the internal anatomical angle of the elbow joint. The actual final angle was determined by each participant according to their own safe and comfortable range of motion. During the experiment, signals were collected and enveloped via EMG sensors with an approximate sampling rate of 1,200 Hz. The signals were processed through normalization and labeling. Each channel was normalized on the basis of its own value range.

**Fig. 4.**
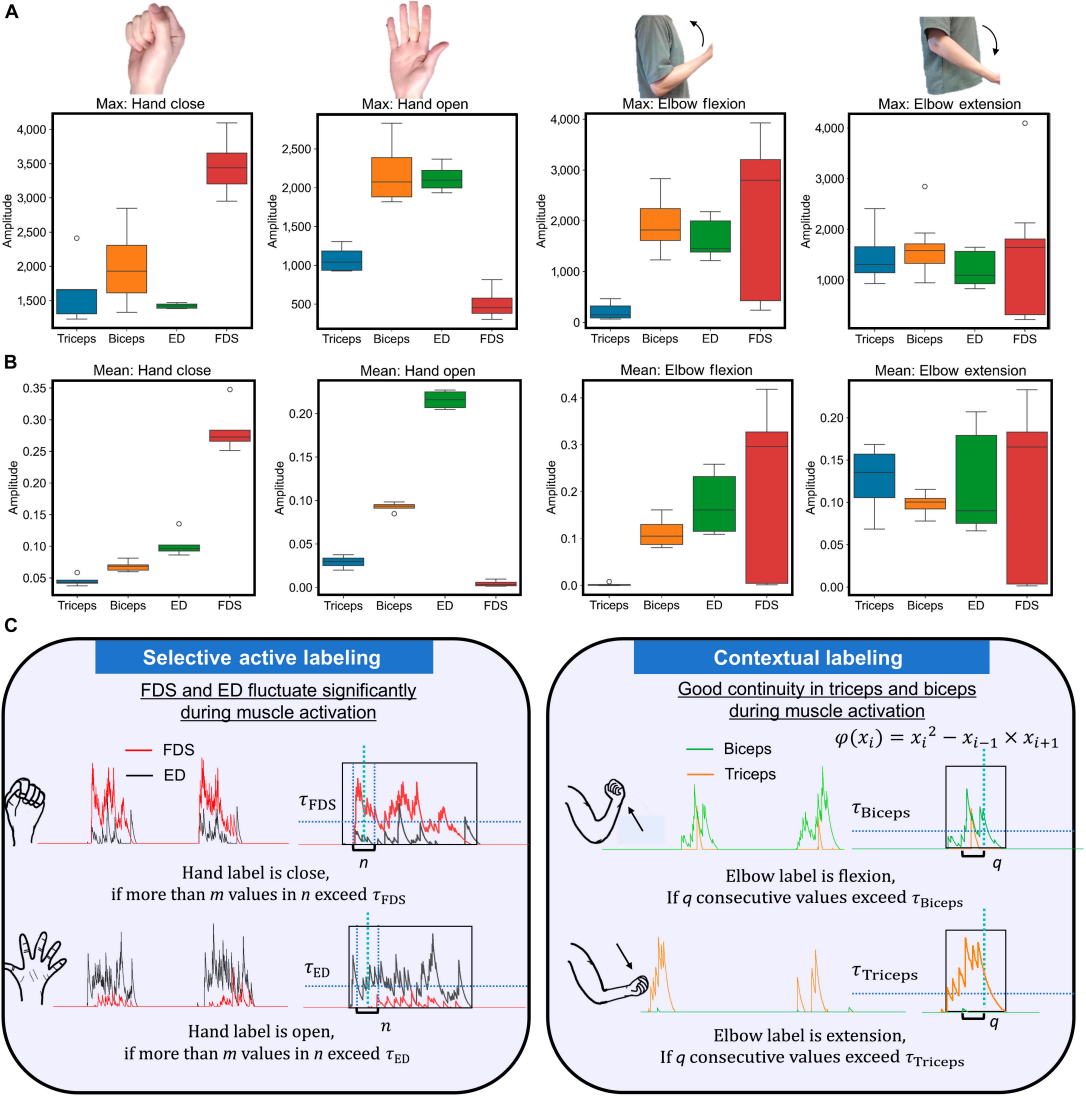
Electromyography (EMG) analysis methods. (A) Maximum values of each EMG channel. (B) Mean values of each EMG channel. (C) Different labeling approaches, including selective active labeling and contextual labeling for hand and elbow data.

Selective active labeling and contextual labeling were subsequently conducted for the hand and elbow states, respectively. First, the maximum value of each EMG channel was calculated and compared, as shown in Fig. 4A, where one box covers the maximum values of different cycles. The range of the maximum EMG value of each muscle is large and varies greatly, indicating that raw EMG amplitudes differ substantially across muscles, motions, and recording periods. Therefore, normalization was applied to reduce the influence of amplitude scale differences on subsequent label generation and feature learning. Moreover, hand- and elbow-state labeling was also necessary in data processing to provide the ground truth for AI model training. To reduce the delay between the EMG information and model labels, thereby enabling timely intent prediction, we labeled the data based on physiological information. The mean value of each channel was calculated and is displayed in Fig. 4B. We found that the performance of each channel is consistent with the principle of muscle exertion. In the closed hand, the FDS is dominant so that its average value is higher than that of the other channels. In contrast, ED is more related to hand opening, in which it has the highest values. Similarly, the biceps and triceps are activated mainly in elbow flexion and extension, respectively.

Accordingly, the selective active labeling and contextual labeling methods are designed based on different signal characteristics (Fig. 4C). In hand labeling, EMG signals rarely remain at a stable high level and instead tend to fluctuate over time. To address this, we propose a selective active labeling approach that assigns labels based on the level of signal activation within a local window. Specifically, the hand state is labeled as close if more than m samples of the FDS exceed a predefined threshold $\tau_{\text{FDS}}$ within a window of size n centered at the current point. The labeling for hand opening follows the same procedure, using the ED signal. In this study, n was set to 500 samples (approximately 0.42 s at a sampling rate of 1,200 Hz), which is consistent with the temporal scale of hand-related muscle activation and matches the model input window length. Using m=0.5n ensures that the assigned labels reflect sufficient activation within the local window, rather than isolated signal spikes. The threshold $\tau_{\mathrm{FDS}}$was determined based on the amplitude distribution of the normalized FDS signal, with only minor subject-specific adjustments when necessary. For the ED channel, $\tau_{\mathrm{ED}}$ was estimated from its mean amplitude during the onset and offset of hand closure, where ED is relatively inactive while FDS dominates.

For elbow muscle labeling, we adopted a contextual labeling strategy, where “context” refers to the temporal continuity constraint imposed on local EMG activation patterns. Compared with hand-related muscles, the EMG activity of the biceps and triceps is relatively continuous during elbow movement. Therefore, instead of using the majority criterion within a long window, we assigned elbow muscle labels only when a continuous sequence of samples met the activation condition. Specifically, elbow flexion was labeled when the biceps signal continuously exceeded its threshold within a window of size q. Elbow extension was labeled in the same way using the triceps signal. In this study, q was set to 250 (approximately 0.21 s at a sampling rate of 1,200 Hz), which allowed for labeling on a finer temporal scale while maintaining consistency with the relatively continuous activation patterns of these muscles. The thresholds for the biceps and triceps were set to 3 times the standard deviation of the corresponding signals in the relaxed state [40], and this continuity criterion was used to assign elbow-state labels only when the local activation pattern remained continuous over a sufficient number of samples. Finally, the generated labels were confirmed in accordance with our data collection protocol to correct a small number of inconsistent labels during movement transitions.

Furthermore, we compared the proposed labeling methods with traditional labeling methods including manual labeling and root mean square (RMS) labeling. Manual labeling is obtained by video observation, which is based on motion completion and may be influenced by visual and judgment errors. The RMS labeling calculates the RMS of the EMG signals within a window to label the middle time of the window. The comparison results are demonstrated in Results.

### Functional level: Modeling for multijoint analysis

#### Deep backbone model development

The characteristics of EMG signals make modeling for EMG analysis challenging. First, EMG signals are easily affected by external noise or cross talk, thereby affecting model recognition effects. Second, muscle activities are nonlinear and nonstationary, which is also shown in EMG signals. Therefore, traditional simple linear signal mapping is inappropriate for EMG analysis. In addition, individual differences also increase the complexity of EMG signal analysis because of differences in muscle structure, neural control, etc. The model is expected to be feasible and applicable for more different users. Finally, EMG signals are high-dimensional time series data with a high acquisition frequency and large amounts of data, which requires efficient computing, especially in real-time application scenarios.

**Fig. 5.**
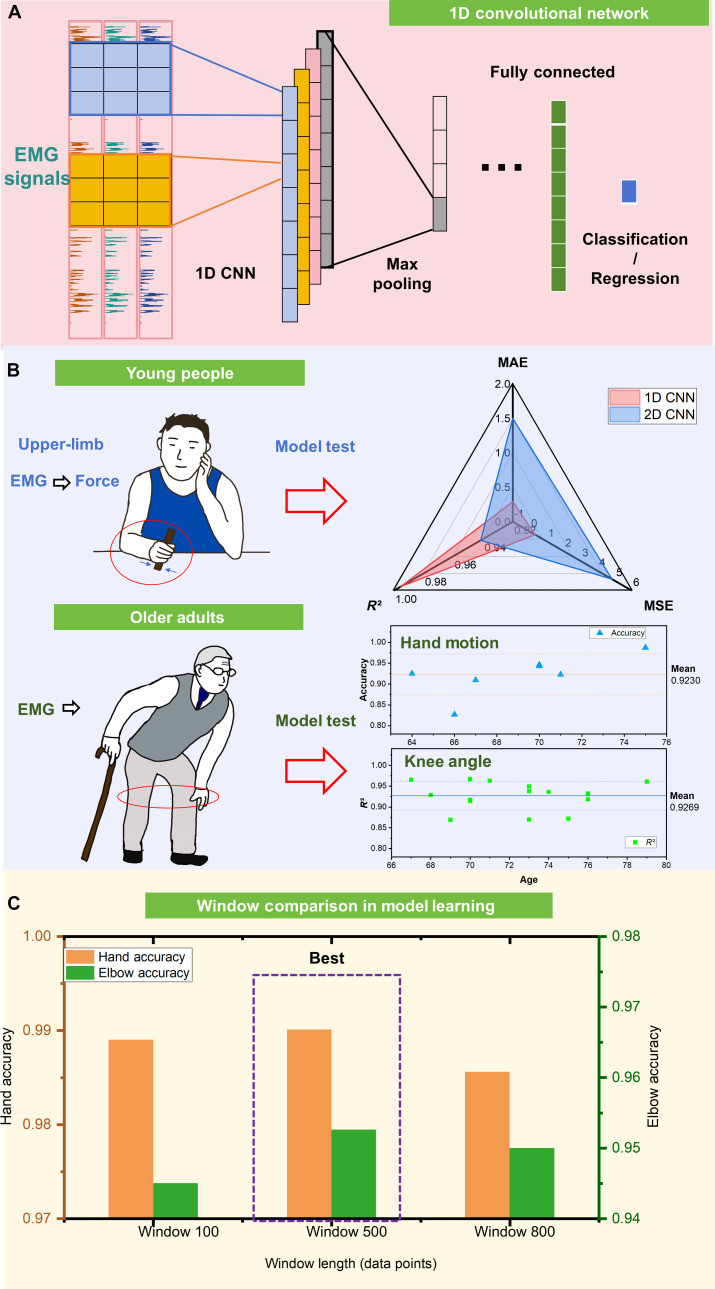
Development of the deep backbone model. (A) Working mechanism of the one-dimensional (1D) convolutional neural network (CNN) in signal analysis. (B) Validation of the 1D CNN model in electromyography (EMG)-based intent prediction in both young people and older adults. (C) Performance comparison of different window lengths in hand and elbow activity prediction.

Convolutional neural networks (CNNs) can effectively identify and extract features from signals. On the one hand, the local perception mechanism enables CNNs to capture critical local features and patterns in signals by convolutional layers. On the other hand, parameter sharing helps address the translation invariance in signals. The multiple superimposed convolutional and pooling layers are able to learn and extract deeper high-level patterns for better recognition performance. To further adapt to EMG sequences, we propose the use of a one-dimensional (1D) CNN to avoid redundant information in the space dimension. Since the 1D CNN considers features in only one dimension (Fig. 5A), it can better capture temporal relationships and local patterns in sequence data. In addition, the computational complexity of a 1D network is relatively low, so it has high computational efficiency when processing large-scale sequence data.

In the preliminary work of model construction, we tested the 1D CNN in EMG analysis for simple and single tasks, as shown in Fig. 5B. Young adults (aged between 20 and 30 years) participated in the EMG-based grip force estimation [18]. The 1D architecture outperforms the 2-dimensional model in the evaluation of the mean absolute error, mean squared error, and coefficient of determination (*R*^2^), indicating that the 1D CNN is indeed capable of extracting useful EMG information from complex EMG signals without tedious handcrafted work. In addition, given that the behavior habits and patterns of older adults can differ from those of young adults, the qualities of EMG signals may be influenced by differences in muscle strength loss, sensory changes, muscle coordination changes, etc.

To verify the effectiveness of our model in older groups, further tests on model predictions of older adults were carried out, as shown in Fig. 5B. We conducted experiments using hand movement data from older adults, including relax, open, and close tasks. Similarly, the 1D CNN was utilized to predict hand motions based on the corresponding EMG signal window. The model achieved high recognition accuracy on these upper-limb data from older adults, with a mean of 92.30%, directly validating its effectiveness in the target upper-limb scenario. In addition, the data of lower-limb EMG signals and dynamic knee angles during sit-to-stand activities were collected from older adults. An average $R^{2}$ of 0.9269 was obtained in their angle prediction. These results suggest that the model can make accurate predictions for elderly people of different ages, whose physical conditions and functions may differ from those of young people. This test further validated the stability and reliability of the deep 1D convolutional model in EMG signal analysis and intentional movement prediction for various subjects.

Based on the preliminary model development, a deep backbone model was designed to extract deep features and map them to different motion states. The sliding-window technique was used to contain enough information in changing EMG signals for continuous recognition. The influence of window length (100, 500, and 800 data points with 5 points as intervals) on offline prediction accuracy is compared in Fig. 5C. The performance at 500 data points (approximately 417 ms) was the best, with accuracies of 0.9901 and 0.9526 for hand and elbow prediction, respectively. When the window length is shortened to 100 data points, the accuracy decreases because of inadequate effective information. When the window length is lengthened to 800 data points, more redundant information is involved, confusing the model to make accurate judgments. With respect to the prediction speed, the window length in this range did not influence the model step speed. At sample lengths of 100, 500, and 800 data points, the average step time for one sample was approximately 15 ms in all cases. Considering the model performance and data complexity, a window length of 500 data points is chosen for our deep backbone model.

#### Model from single-joint prediction to multijoint coordination

**Fig. 6.**
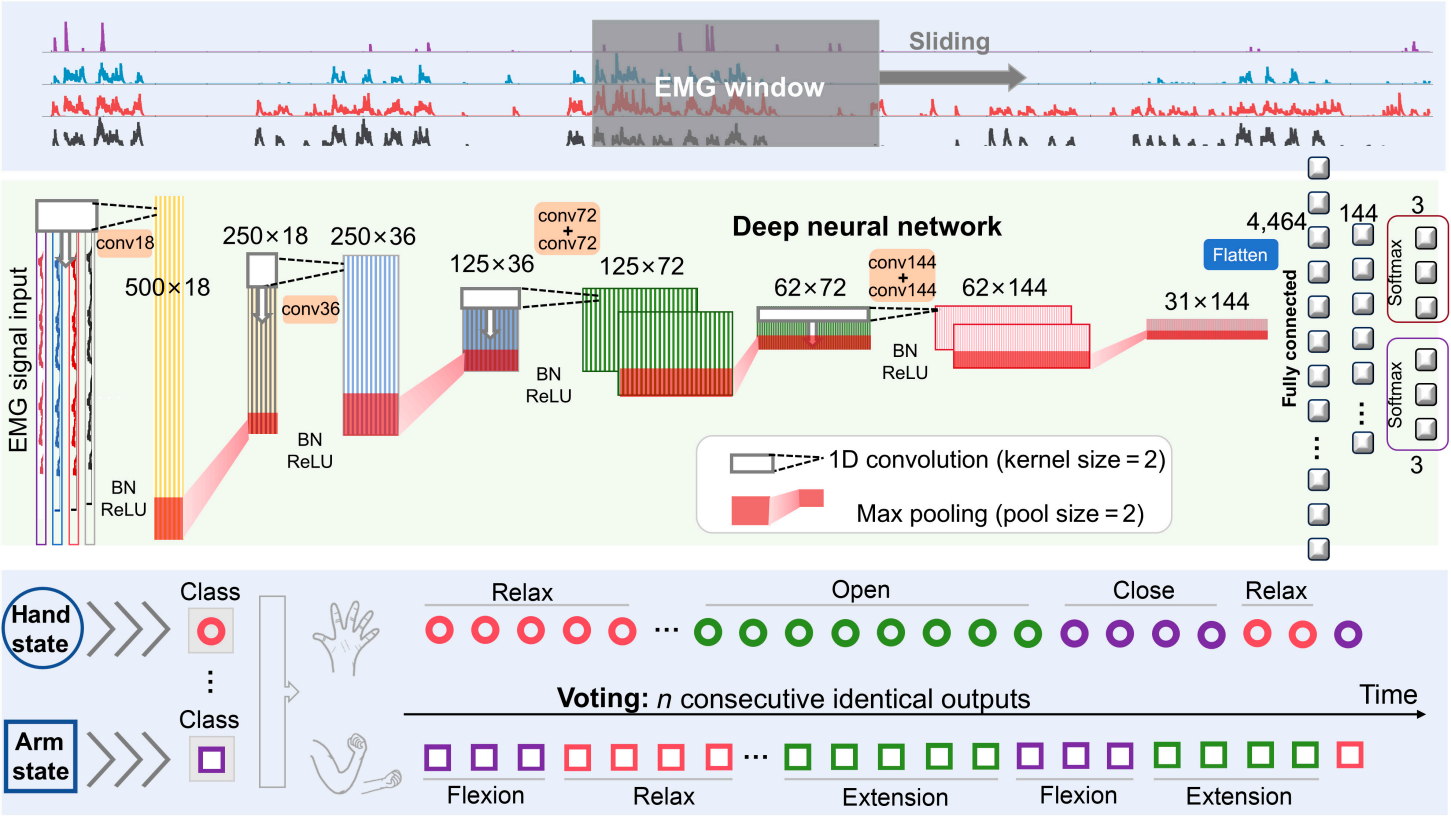
Functional level: real-time data processing and intelligent recognition through a multitask deep learning model and a voting technique.

At the functional level, most daily activities require the coordination of multiple joints, and AI predictions of simultaneous movements of both hands and elbows on the basis of collected 4-channel EMG signals are studied and demonstrated. In joint coordination, the muscles surrounding joints must contract and relax in a synchronized manner to generate the necessary force and maintain stability, which is reflected in more complicated EMG signals and cross talk. Based on the preliminary work, we design a compact deep backbone model for intelligent and efficient intent prediction (see Fig. 6). The processed 4-channel EMG window serves as the model input. In the front part of the model, features are extracted by 1D convolutional layers where the hand and elbow predictions share the same structure and weights. The first convolutional layer, which includes eighteen 1D convolutional kernels (the kernel size is 2), extracts preliminary EMG features. ReLU activation is used to alleviate the vanishing gradient problem and accelerate model convergence. L2 regularization is introduced to avoid overfitting. We also utilized batch normalization to improve model generalizability. Then, a max pooling layer with a pooling size of 2 is constructed to further filter information to keep the most salient features of each region in the feature map. After that, a similar 1D-convolution-max-pooling structure with 36 kernels is designed. In addition, 2 continuous convolutional layers with 72 kernels followed by a max pooling layer further extract deep effective features. A similar structure with 144 kernels is connected to it. The final extracted feature maps are flattened and then converted to a fully connected layer with 144 elements. To accomplish the task of 2 joint predictions, 2 fully connected layers are separately connected to the last layer, and each has 3 elements with softmax activation to output probabilities of hand/elbow motion states.

An algorithm based on a voting mechanism was designed to reduce the fluctuations in model output, outlined as follows: A sliding-window queue is created, and its maximum length is specified. For each new model output value, the following steps are performed: (a) Check window length: The sliding-window queue is checked to determine if it has reached its maximum length. (b) Voting mechanism: A voting window of 15 data points is used. If the window is full, all values in the queue are compared with the current output value. The latter is the voted result if all values are identical to the current output value. Otherwise, the previous voted result is retained. (c) Window update: If the window is full, the oldest value in the queue is removed. Otherwise, the model’s current output value is added to the end of the queue until the window reaches its full length of 15 data points. The fixed-window voting rule is used as a deterministic postprocessing step to suppress sudden prediction jumps in the output sequence.

### Behavioral level: Adaptation to ADLs

**Fig. 7.**
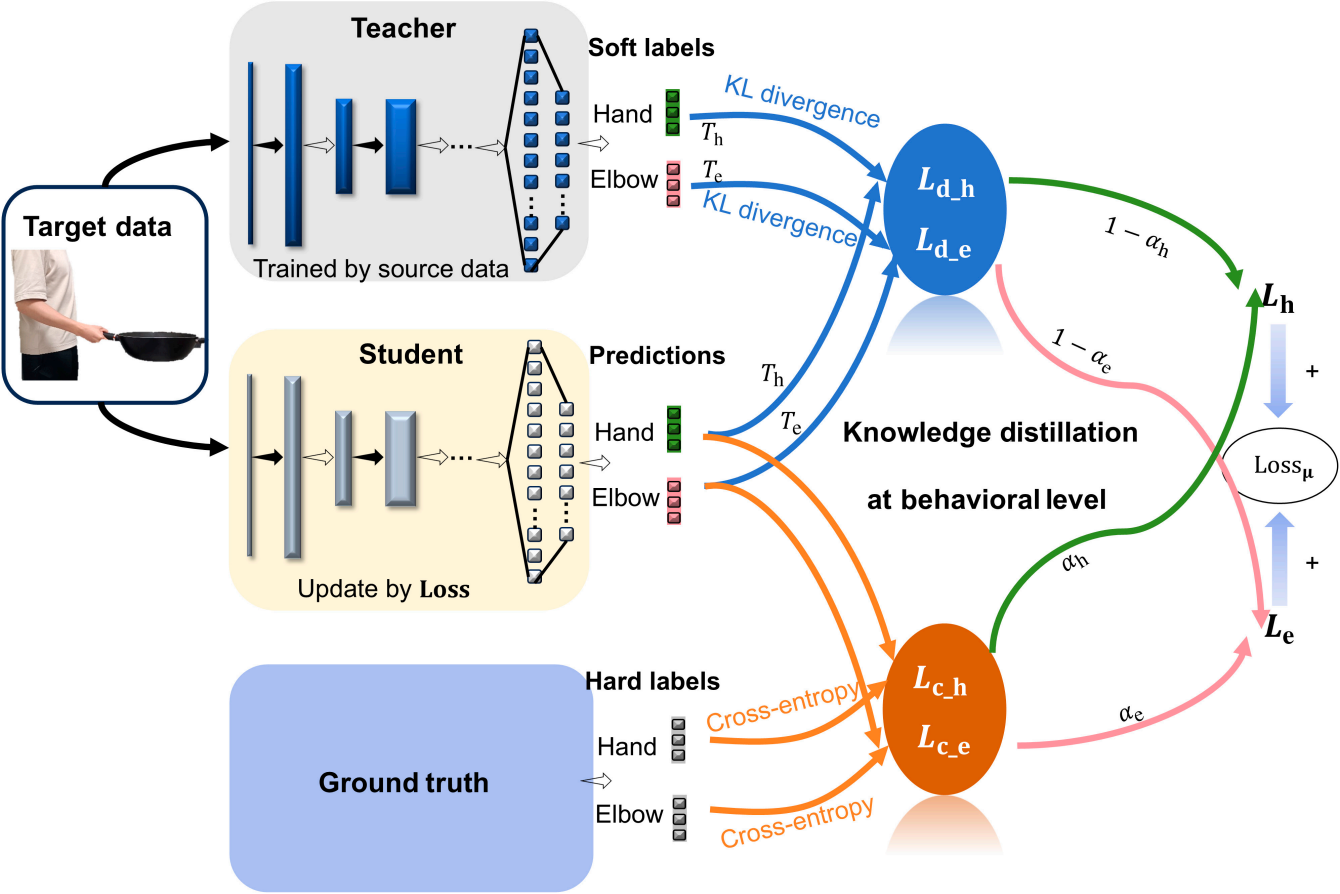
At the behavioral level, knowledge distillation is used to absorb new knowledge to generalize ability in both hand and elbow prediction from fundamental movements to complex daily activities.

Although fundamental joint movements are basic needs in daily life, ADLs include more complex behavioral actions, so advancement of the adaptation of the model to practical complex activities is required to facilitate convenient and independent daily living. Here, at the behavioral level, for more challenging daily activities, we propose an approach using knowledge distillation to inherit the learned knowledge from the functional level and increase knowledge to the behavioral level, as shown in Fig. 7.

We take incorporating cooking knowledge as an example. The new knowledge of cooking is defined as new target data, in which we collect picking up a wok and moving it around. The EMG information during cooking is different from that during functional joint movements because of different gestures and ways of exerting force. To compensate for the incomplete elbow-extension information and prevent forgetting, a small amount of hand-open data with elbow extension and flexion was included in the target data, ensuring that this motion is not omitted during distillation. The original trained model at the functional level is defined as the teacher, which is trained through basic joint motions as source data. A student model with the same architecture as the teacher is constructed for new training. The student is updated with soft labels and hard labels from the teacher model.

The model is optimized to inherit the functional knowledge and then learn new actions (such as picking up and moving a wok) with only a small amount of cooking data. During knowledge distillation, the weights of the teacher model do not change. In contrast, the weights of the student model are updated on the basis of the average sum (${\text{Loss}}_{\mu }$) of the hand prediction loss ($L_{h}$) and elbow prediction loss ($L_{e}$). Taking the hand as an example, $L_{h}$ is obtained by the weighted sum of student loss ($L_{c\_h}$) and distillation loss ($L_{d\_h}$). $L_{c\_h}$ is calculated using cross-entropy to compare the student predictions and the ground truth (hard labels) on the basis of the target data. $L_{d\_h}$ can be computed via Kullback–Leibler divergence loss to reflect the distance between the distributions of student soft predictions and teacher predictions (soft labels), with a distillation temperature to adjust the smoothness of the prediction distribution. In the training process, the temperature and ratio between the distillation loss and student loss are the most important hyperparameters. A higher temperature leads to a smoother distribution, making it easier for students to learn soft knowledge from the teacher. In addition, with a higher proportion of distillation loss, the student model will learn more about new knowledge. However, if it is too high, the mastery of old knowledge decreases. Taking reducing overall loss as the training goal, the student can not only retain the 2-joint coordination knowledge from the teacher but also learn cooking operations in daily life. This approach enables the accumulation of knowledge, thereby avoiding the problem of learning from scratch, and has the flexibility to accept new data input at any time.

### Real-time control system construction

To verify the effectiveness of our approach in real-life applications, we constructed a real-time control system consisting of 3 main parts: sensors, an edge computing unit, and a robot. During upper-limb activities, EMG signals are acquired by the EMG sensing hardware (AD623ARMZ, TLC2274, and TLC2272) together with a microprocessor (ESP32-WROOM-32) and are continuously transmitted to the Nvidia Jetson Nano Developer Kit (69 mm × 45 mm) through a serial interface at 115200 baudrate. On Jetson Nano, the real-time control program was implemented in Python 3.6 using TensorFlow 2.6.0/Keras. The trained dual-output AI model was deployed on Jetson Nano by reconstructing the network architecture and loading the trained weights for online inference. In the deployed system, a sliding window containing 500 time steps and 4 EMG channels was used as the model input for simultaneous prediction of hand and elbow states.

Before system operation, a simple joint motion calibration is performed, and each EMG signal channel is normalized using the maximum value of each channel. During continuous operation, when a channel experiences a higher peak value, the calibration maximum value is updated to maintain signal normalization across different muscle activation levels. To support continuous real-time operation, the Jetson Nano implementation is organized into concurrent processes for serial data reception/processing and model inference/control output, respectively. The predicted hand and elbow states are further stabilized using a voting strategy before being encoded into control commands and transmitted via Bluetooth to the 6-axis collaborative robotic arm (myCobot 280, Elephant Robotics), which then executes the corresponding joint movements.

To improve practical deployment efficiency, we employed a lightweight inference pipeline optimized with preallocated memory tensors for online prediction on Jetson Nano. The online responsiveness was evaluated using time-stamped runtime logs that recorded the complete online process, including serial input data, model outputs, and control signals. Based on these logs, the optimized implementation achieved an average per-cycle latency of approximately 27 ms, substantially faster than the 110 ms using direct model prediction calls. This real-time implementation demonstrates the practical feasibility of the proposed framework for embedded real-time upper-limb control.

## Results

### Physiological level: EMG labeling schemes

**Fig. 8.**
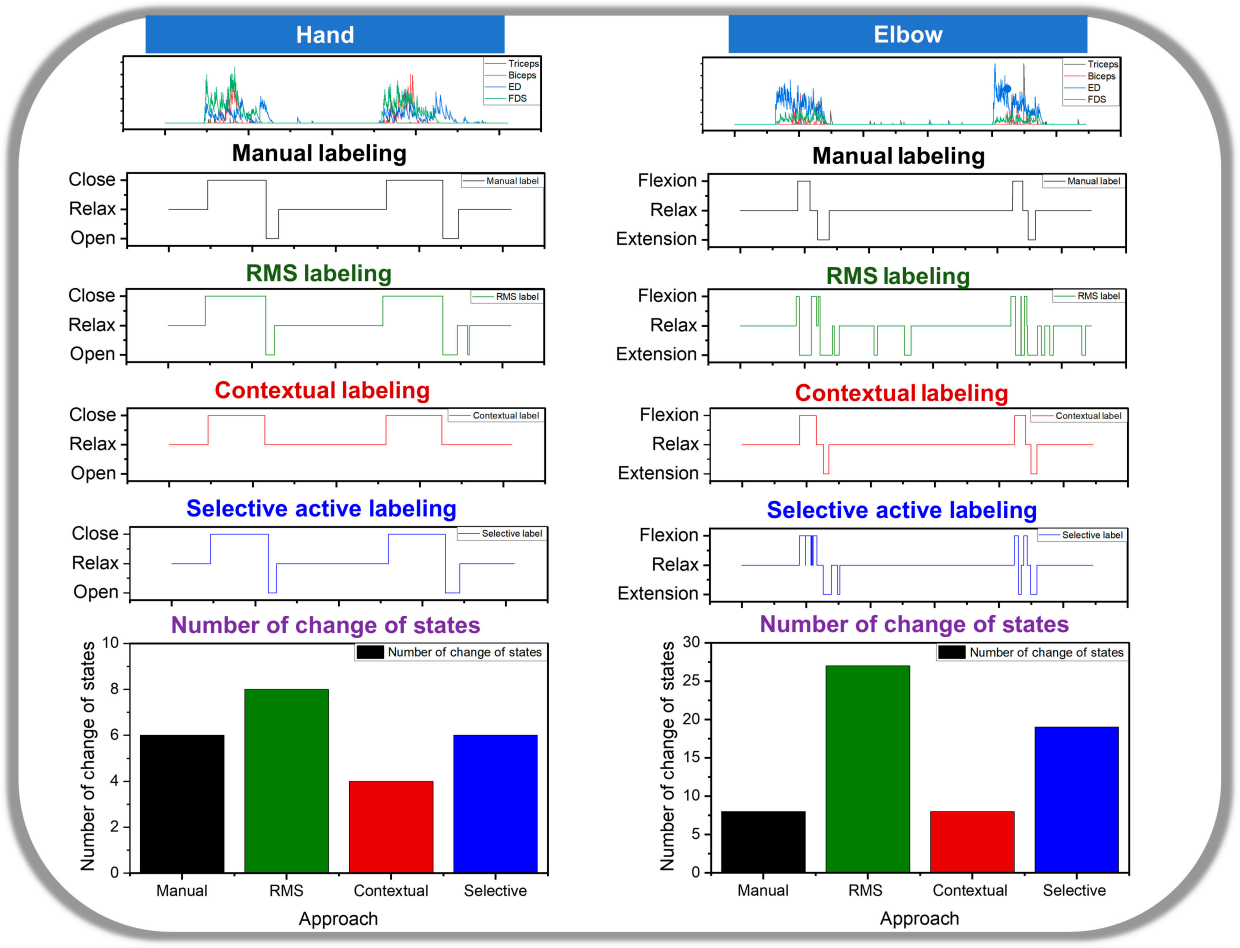
Comparison of the results of different labeling approaches.

As shown in Fig. 8, we selected a representative data segment containing different hand and elbow movements and compared the results of different labeling methods. In addition to visual comparison of the annotation curves, we also conducted a quantitative comparison using the number of change of states as an evaluation metric.

In hand labeling, selective active labeling was most consistent with manual labeling in terms of the number of state changes. Specifically, manual labeling showed 6 state changes, while RMS labeling showed 8, contextual labeling 4, and selective active labeling 6. This result indicates that selective active labeling matches the counting results of manual labeling, while RMS labeling introduces additional state transitions, and contextual labeling produces fewer state transitions than manual labeling. Therefore, selective active labeling is more suitable for situations with short activation durations or large signal changes during activation. RMS labeling tends to shorten the detected activation time, while contextual labeling may miss some important activation information.

In elbow labeling, contextual labeling was closest to the results of manual labeling. Manual labeling involved 8 state transitions, while RMS labeling involved 27, contextual labeling involved 8, and selective active labeling involved 19. This quantitative comparison shows that contextual labeling and manual labeling have completely consistent counts, while RMS labeling and selective active labeling produce more frequent label transitions. Therefore, contextual labeling can make more accurate judgments when the relevant signals have better continuity, while other methods are more susceptible to irrelevant signals and tend to change labels unnecessarily.

The results indicate that the proposed intelligent labeling methods are effective in that they can be applied based on different signal characteristics to provide precise learning information for the AI model. Although the double confirmation of labels is necessary to eliminate some fluctuations, compared with conventional manual labeling, the load of recording data via other sensors or cameras and the work of data collection and analysis for labeling could be greatly improved via our proposed labeling methods, which are based on physiological conditions.

### Functional level: Offline and online multijoint model performance

**Fig. 9.**
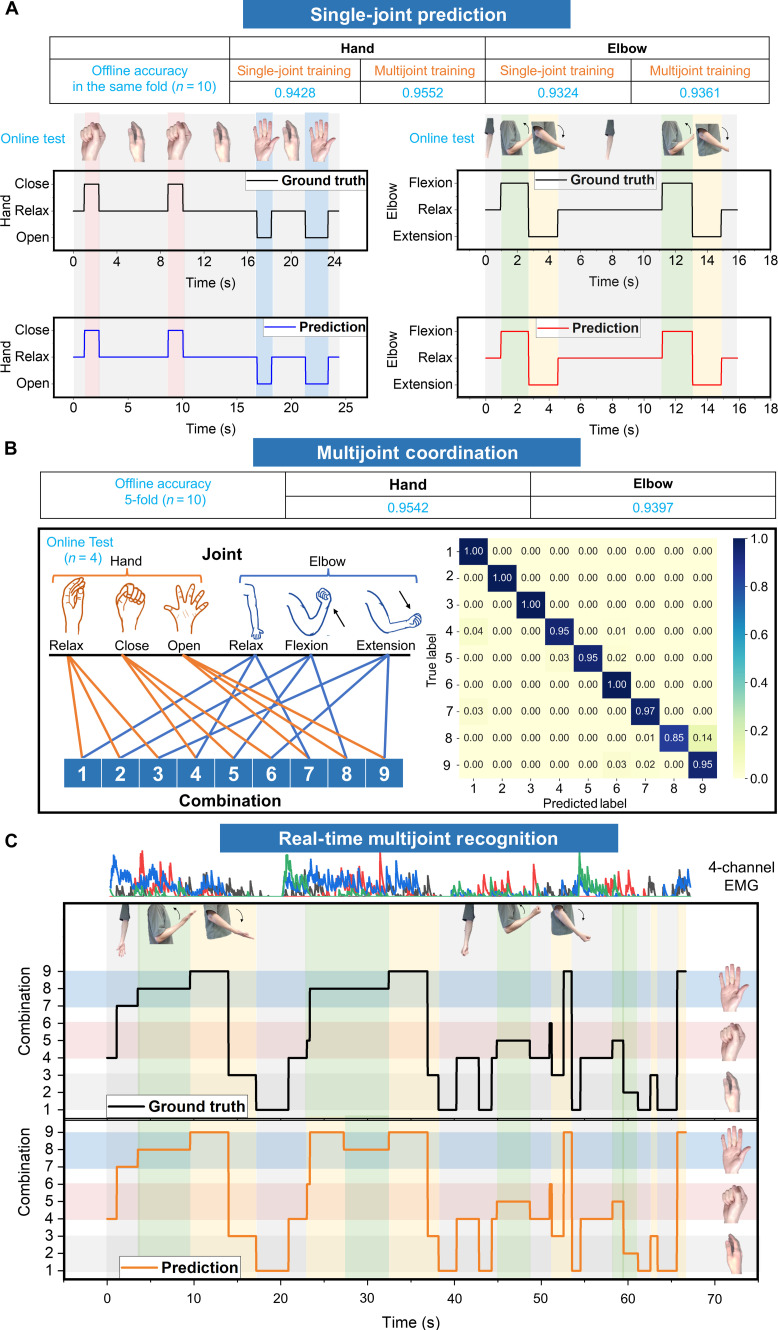
Performance of single-joint prediction and multijoint coordination at the functional level. (A) Single-joint predictions of the hand and elbow. (B) Combination configuration and confusion matrix. (C) Real-time comparison of multijoint coordination between the ground truth and model prediction.

Based on our deep learning model, we carried out offline and online tests to prove the model potency from single-joint prediction to multijoint coordination. First, we compared the performance within the same fold of the single-joint model and the multijoint model, as shown in Fig. 9A. In single-joint training, hand states are predicted on the basis of the relevant muscles of the ED and FDS as the input, whereas elbow states are estimated on the basis of the triceps and biceps as the input. The multijoint model not only integrates multiple types of information but also strengthens the efficacy of predictions. The elbow movement prediction accuracy slightly increased from 93.24% to 93.61%, whereas the hand movement prediction showed a more pronounced improvement, increasing from 94.28% to 95.52%. The integration upgrades the comprehensive performance. There are many reasons that the related 2 tasks help each other in information learning, opportunities for information and feature interactions are provided during this process, and model generalization can be optimized. Overall, multijoint modeling has shown superiority over single-joint prediction.

In offline testing, we further conducted a 5-fold cross-validation. The prediction accuracies for hand and elbow movements reached 95.42% and 93.97%, respectively. We also compared our model with 2 representative baseline models suitable for online EMG analysis: a random forest classifier based on handcrafted EMG features and a temporal model based on a long short-term memory network using raw EMG windows as input. All models were evaluated under the same experimental settings using 5-fold cross-validation. The proposed backbone model achieved an optimal balance between classification performance and computational efficiency. Detailed settings and results are provided in the Supplementary Materials.

Furthermore, we conducted real-time experiments to verify its practical capability for both single and multiple joints, as shown in Fig. 9. Each joint movement was performed once in calibration before the test to normalize the range of the EMG. Throughout the test, the calibration can be continually updated if there is a higher peak in a certain EMG channel. In single-joint prediction, consecutive hand/elbow movements are accurately predicted (Fig. 9A). For example, hand closing and opening are performed twice. The prediction results below demonstrate the synchronized model predictions. Similarly, the AI model yields good prediction results for elbow joint individuals. With the change in single-joint movements, the model can accurately predict the individual joint states. The model performance for a single joint is excellent, and incorrect predictions rarely occur.

In addition, functional upper-limb activities require more complicated joint coordination. As shown in Fig. 9B, a total of 9 combinations of hand–elbow coordination are composed and numbered. For example, hand closing with elbow flexion is combination 5. In the real-time test, participants are asked to perform hand closing with elbow flexion and extension twice and then perform hand opening with elbow flexion and extension twice. Some natural movements are performed among transforms in different combinations. With the 4-channel EMG signals as the input, our model automatically learned effective patterns, translated them to accurate probabilities, and continuously made judgments on both hand and elbow states. The robot was controlled by the voted predictions based on myoelectric signals simultaneously.

Considering consecutive output values within the sliding window reduces the impact of outliers or noise on the final result, and a relatively stable output is achieved. An example of a complete process is shown in Fig. 9C. Our model predicted 2 tasks, including both hand and elbow movements, based on 4-channel EMG signals. The elbow states are labeled on the top: gray, green, and yellow blocks represent elbow relax, flexion, and extension, respectively. The hand states are marked on the right: gray, pink, and blue blocks represent hand relax, close, and open, respectively. Different cross overlapping areas correspond to different combinations. To describe the movements clearly, the *y*-axis represents combinations, which is consistent with the settings in Fig. 9B. The black curve shows the ground truth of the participant activities, whereas the orange curve represents the model predictions in real time. In general, the predictions fit the true movements very well in the 2-cycle real-time test. Some major errors occur in the motion transform periods because of the unstable EMG signals. For example, the model mistakenly recognized combinations 8 and 9 in the hand-open elbow transform at approximately 24 s. More detailed information can be found in the accuracy confusion matrix (see Fig. 9B). The accuracies of hand-relax elbow movements, including combinations 1 to 3, reach 1. Hand close and hand open with elbow relax (combinations 4 and 7) are more likely to be identified as hand-relax elbow relax (combination 1). This is because sometimes, a human subject can keep their hand close/open without force, and myoelectric activation is very weak, which may confuse model predictions. In addition, some hands close/open with elbow flexion (combinations 5 and 8) are mistakenly recognized as elbow relax, because the model is not particularly responsive to situations involving extremely low muscle activation. Sometimes, the biceps do not contract a lot at the beginning of elbow movements. In addition, sometimes elbow flexion and extension are confused, especially when the hand is open (combinations 8 and 9), because the triceps are more prone to being activated when the hand is open. Overall, multijoint coordination, including 9 combinations of hand and elbow movements, can be predicted using our proposed AI model in real time with a high accuracy of 95.34% based on myoelectric information, enabling precise neural-driven robot control at the functional level. A video showing the real-time control process can be found in the Supplementary Materials.

### Behavioral level: Simple ADL performance and adaptation to complex ADLs

**Fig. 10.**
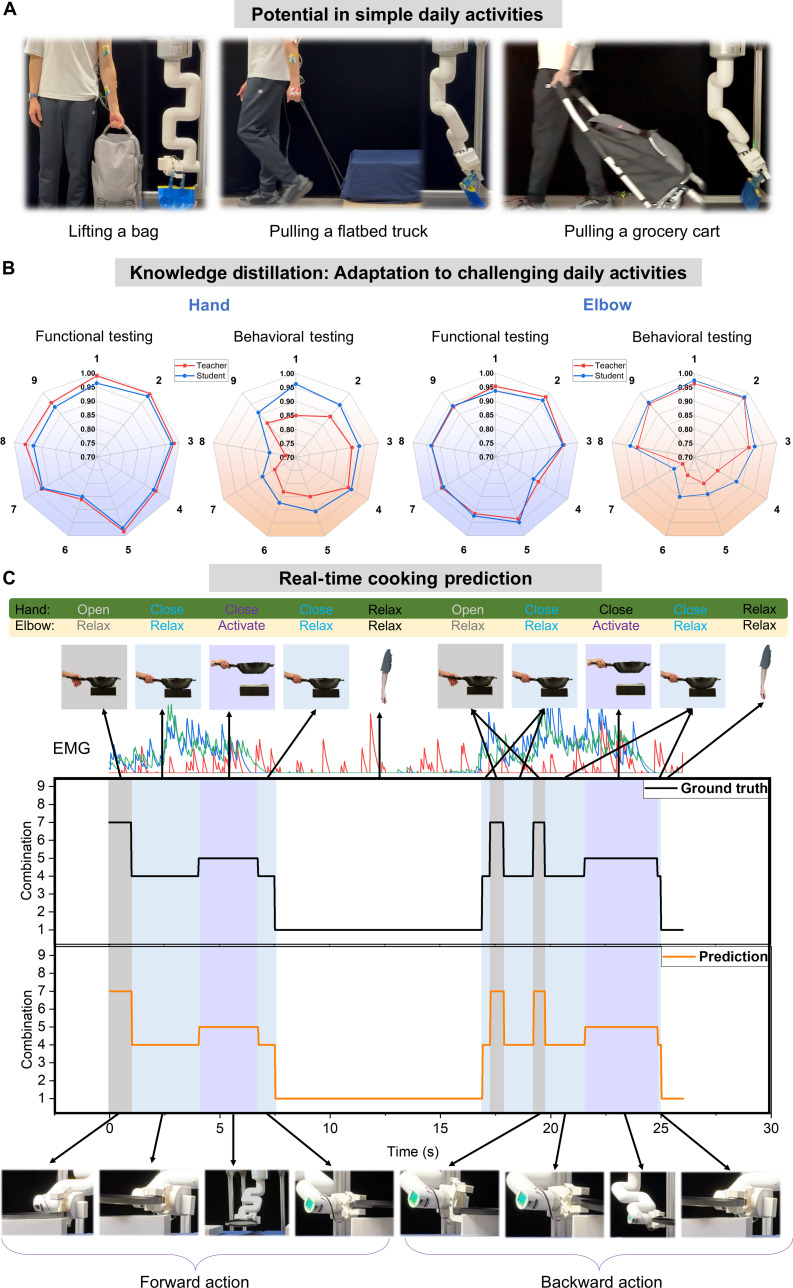
Behavioral performance of application in daily activities. (A) Electromyography (EMG)-driven multijoint control tests in simple scenarios in daily life. (B) Adaptation to challenging daily activities via knowledge distillation. The performance of the teacher model and that of the student model in functional joint coordination and behavioral cooking activities were compared. (C) Real-time comparison of cooking between the ground truth and student model predictions.

Tests in several simple daily scenarios (Fig. 10A) were carried out. On the basis of upper-limb EMG signals, activities such as lifting a bag, pulling a flatbed truck, and pulling a grocery cart can be assisted via intent prediction. A video can be found in the Supplementary Materials showing the real-time dynamic control process. Although both the hand and elbow are involved in these activities, the corresponding force generation patterns and muscle activation modes differ substantially. Moreover, we found that the functional-level model is insufficient to stably handle these different scenarios. In lifting, the participant mainly overcame the bag weight. In truck pulling, friction between the wheels and the ground hinders upper-limb movement. In cart pulling, both friction and cart weight can lead to the activation of hand- and elbow-related muscles. The realization of these various applications further highlights the potential of our approach in intelligent assistance for more practical daily scenarios.

To further adapt to complex ADLs, we proposed a distillation approach and applied it in a cooking task, in which both the hand and elbow are activated, and this task has higher uncertainty and continuous switching compared to functional-level movements. Here, using knowledge distillation, the student model was obtained. We compared the performance of the student model with that of the teacher model. An elderly person was unable to complete the experiment; therefore, *n* = 9. As shown in Fig. 10B, the student model has higher accuracy than the teacher model in cooking. In terms of hand action prediction, accuracies increased by 1.33% to 11.25% across all participants. In particular, the accuracy of subject 1 improved from 84.95% to 96.20%. Notably, catastrophic forgetting did not occur with the guidance of the teacher. In elbow movement prediction, the student performance enhanced by 0.34% to 8.2% in cooking. The student still maintained good performance in terms of the teacher’s knowledge. The change in the testing accuracies is less than 1.94%. Generally, in behavioral testing, the improvement in student accuracy is more pronounced when the teacher accuracy is relatively low. In functional testing, while the student model sometimes experiences performance degradation, it is small and still maintains a high level. Hence, when new related knowledge is encountered, the original models, which are not good at adapting old knowledge to new applications, are in greater need of knowledge distillation. In addition to absorbing new knowledge, the student model can also inherit the original knowledge of fundamental joint combinations following the advice of the teacher. A more knowledgeable and adaptive model with better generalizability can be obtained using knowledge distillation.

With the distillated student model, we carried out real-time experiments on cooking (see Fig. 10C). The continuously changed hand and elbow states are labeled on the top of the curves. The black and orange curves represent the ground truth and model prediction, respectively. The robot was controlled in real time on the basis of the model prediction. A video can be found in the Supplementary Materials showing the whole process of real-time behavioral testing of adaptation to cooking.

At the beginning of the test, the participant closed his hand to grasp the handle while the elbow was relaxing. The color change from gray to blue illustrates this process. Then, picking up the wok and moving it from one side to another side were performed. The hand and elbow were both activated in this purple state. Finally, the participant put down the wok, as shown in the blue block. The model prediction accurately followed the change in the ground truth, demonstrating strong knowledge proficiency. After the hand and elbow relaxed, the participant moved the wok back. During this process, he grasped and released the handle twice, when the student model also proved its reliability and robustness. The prediction changed in time with the change in user intent.

While cooking scenarios serve as a typical case study at the behavioral level, ADLs involve interaction patterns far beyond manipulating cooking utensils. To broaden the task coverage, we also applied the proposed function–behavior distillation method to other ADL-related tasks, such as lifting a bag and pulling a grocery cart. Compared to cooking, these tasks introduce different object properties and motion requirements. Detailed experimental results are available in the Supplementary Materials, further demonstrating that the proposed framework can be extended to a wider range of goals oriented toward ADLs.

## Discussion

Recent related studies have shown growing interest in sensor-based and intelligent assistive systems for supporting daily living activities among the elderly [41–44]. In this context, active motor assistance for daily activities, particularly coordinated multijoint support, has emerged as an important research direction. The analysis of the correlation between human intent and human activities has been an area of extensive research for many years, as it provides an important basis for intention-driven assistive control. People strive to find effective methods in this field, but there are still challenges such as complex manual work, signal instability, and scenario variations [45,46]. To overcome these challenges, in this work, we propose realizing intent prediction and application systematically at 3 levels: the physiological, functional, and behavioral levels. Based on the correlations and differences among these levels, a novel AI-driven multijoint EMG-control approach incorporating myoelectric signal adaptability was designed and implemented in a coordinated hand–elbow robotic control system. Studying different levels enhances the discovery of signal-to-control relationships from simple actions to complex human motions. At the physiological level, different labeling methods are designed for different joints based on relevant muscle activities, successfully reducing the manual workload in traditional processing and maintaining the accuracy and effectiveness of information in data labeling. At the functional level, not only single-joint movements but also joint coordination is estimated via our deep learning model and voting technique, which is trained by fundamental joint tasks. At the behavioral level, when encountering new activities, the model can absorb new knowledge through knowledge distillation without having to be retrained using all of the data. Overall, the realization of 3 levels verified the effectiveness and reliability of our system for assisting robots, which has high potential for enabling independent elderly people’s daily living.

We have overcome challenges from the physiological level in efforts to jump from theory to real-life applications in EMG-based control. First, tedious and inefficient labeling for different muscle motions has been a challenge in mapping myoelectric information to specific motions. Traditionally, extra sensors are usually required to wear or set to monitor motions during EMG signal collection. For example, a camera-based motion capture system was used to record lower-limb motions [47]. The experimenter utilized a key fob to label the motion intent of the subject in the benchmark dataset ENABL3S [48]. In our study, we labeled EMG signals at the physiological level for fundamental joint movements. Two different labeling approaches, selective active labeling and contextual labeling, were designed to address situations with different muscle activities effectively. Comparison experiments were carried out, and the effectiveness of these methods was further validated. In this way, the labeling of the ground truth no longer depends on extra sensor recording. The load on the user can be reduced, and the work for the developer can be simplified. Second, the fluctuating and unpredictable signal amplitude range is also recognized as a considerable concern. If the EMG signal range is not standardized every time, the model cannot make a fair judgment in different signal situations. To address this issue, we propose simply performing basic joint movements for calibration and normalizing the range of each EMG channel using the maximum value of each channel before executing the system. With this approach, the system is still reliable a few months after data collection. Overall, these designs have effectively overcome the challenges and difficulties at the physiological level from myoelectric information to reliable functional applications.

We predicted multijoint intent to expand the application scenarios and benefit both targets at the functional level. Although it is challenging, myoelectric recognition for multiple-joint coordination is necessary for users to complete daily activities in real life [36]. Although some studies have noted the importance of multiple joints in EMG information decoding, most have concentrated on separate joint recognition. For example, 4 hand gestures, 2 wrist motions, and 2 kinds of grasping have been recognized via a transient EMG classifier [49]. Similarly, in addition to grasping gestures and rested states, only one contraction state was estimated in an EMG-driven dexterous robot [50]. Our approach is based on a fundamental single joint and aims to output multijoint coordination intent. Different joint combinations and expansions in coordinated real-life practice are performed. A multioutput AI model using a 1D CNN for feature extraction has been constructed. The hand and elbow predictions share the same weights in the convolutional layers, followed by separate fully connected layers for each task. The abundant intent information can compensate for robot control. In addition, the results suggest that the performance of a single task is enhanced when multiple types of joint information are learned. This is because opportunities for sharing features are created, proofs of redundant information are strengthened, and model generalization is improved. Moreover, we noticed that unstable EMG signals easily caused inevitable fluctuations in the prediction results. Although the influence of such a phenomenon on accuracy is slight, sudden system changes are dangerous and unpredictable. To address this, we introduce an adjustable voting technique to smooth the model output, thereby ensuring more stable predicted values and the robustness of real-time applications.

Traditionally, when encountering a new task, we either train a large model using all of the data or train one more model with new data. Although researchers have proposed the use of transfer learning for new users, they tend to include as many gestures as possible in their dataset [51]. However, such methods require more time and computational resources for training. Inspired by how humans learn to use tools, if the model is likely to master the knowledge of complex activities on the basis of the knowledge of fundamental joint movements, a more efficient and scalable system can be obtained. For example, efforts have been made, and 73.36% accuracy has been achieved in new hand gesture estimation using few-shot learning [52]. In our work, considering that complex coordinated daily activities can be disassembled into fundamental action groups, we designed an approach using knowledge distillation to enhance myoelectric signal adaptability in new tasks. Taking cooking activities as an example, both the hand and elbow are activated during cooking, although the EMG patterns associated with cooking are different from those associated with joint functional movements. Knowledge distillation is commonly used to transfer knowledge from a large model (teacher model) to a smaller model (student model) [53,54]. Recently, SD has been introduced for knowledge transfer, in which the structures of the teacher and the student networks share the same architecture and knowledge distillation is used to absorb more different types of knowledge [55–57]. The model acts as both a teacher and a student, using its own knowledge to refine its understanding and generalization capabilities, thereby improving performance. Here, we use SD to inherit teacher knowledge by matching the soft targets generated by the teacher and learn new cooking knowledge by matching hard targets produced by the student on the basis of new cooking input data. Therefore, knowledge distillation can help generalize the teacher model’s knowledge to new tasks without forgetting the original skills. In this way, when new data are incorporated, the model can refine their weights to adapt to changing patterns in the data. The model can also exhibit greater resilience and robustness, enabling it to handle noisy data, outliers, and inconsistencies in the data. Compared with the original teacher model, the student model using knowledge distillation exhibited higher accuracies in both hand and elbow activation during the cooking process. The results indicate the effectiveness and efficiency of our novel method for adapting to new complex daily activities, providing the possibility to expand more practical application scenarios in myoelectric fields.

The overall contribution of this work lies in providing a systematic solution for EMG-based multijoint coordination. The framework proposed in this paper considers the entire path from physiological signal processing to functional intent decoding and behavioral adaptation during daily activities. Within this path, model distillation serves as a bridge between functional intent decoding and behavioral adaptation, allowing the model to incorporate ADL-specific knowledge while retaining the functional capabilities learned from basic actions. This integrated perspective is particularly important for the development of next-generation assistive robotic systems, as practical usability depends not only on decoding performance under controlled experimental conditions but also on adaptability, stability, and compatibility with real-world motor tasks. More importantly, this study demonstrates that EMG-driven assistance should not be viewed merely as an intention recognition problem, but rather as a multilayered human–robot collaboration problem. Our proposed framework not only provides a technical approach for coordinated upper-limb assistance but also lays a broader foundation for the design of practical and adaptive assistive systems in the future.

Future research should further explore the following directions: First, flexible sensing technology could be integrated into the acquisition system to improve wearing comfort, ease of use, and long-term applicability in everyday scenarios. Compared to traditional electrode arrangements, flexible sensors can better conform to the skin, reducing the burden on users during prolonged operation. Second, although current systems have achieved online inference and robotic execution on embedded edge devices, the overall response speed is affected not only by model inference time but also by signal buffering, communication overhead, and output stability. Therefore, future work will further optimize model lightweighting and system-level latency to enhance real-time response capabilities in practical deployments. Third, from a human–robot interaction perspective, more interaction mechanisms should be introduced to improve the intuitiveness and usability of the system, such as user feedback fusion. These efforts will further enhance the translational potential of the proposed framework and support its application in practical assistive and rehabilitation environments.

## Conclusion

In summary, this study developed a coordinated hand–elbow myoelectric assistive strategy within a systematic framework spanning the physiological, functional, and behavioral levels. To support multijoint assistance in daily activities, we addressed EMG analysis challenges, designed physiologically informed labeling methods, developed a deep backbone model for multijoint intention decoding, and incorporated knowledge distillation techniques to improve adaptability to new task scenarios. The proposed framework achieved an overall classification accuracy of 95.34% across 9 motion combinations in real time, while the knowledge-distilled model improved prediction performance by up to 11.25% in new tasks. Furthermore, real-time robotic control experiments involving joint coordination and different new daily activities validated the system’s stability and reliability. These findings demonstrate that effective EMG-based multijoint assistive systems depend not only on accurate intention decoding but also on robust adaptation to real-world behavioral contexts. Overall, this work provides an integrated technical route for coordinated upper-limb assistance and offers useful guidance for the development of assistive robotic systems for daily use, thereby potentially contributing to improved independence and quality of life for older adults.

## Ethical Approval

The study was conducted in accordance with the Code of Ethics of the World Medical Association (Declaration of Helsinki). Ethical and experimental procedures and protocols were approved by the City University of Hong Kong (Ethics Approval Number: H003131) and The University of Hong Kong (Ethics Approval Number: EA1903040).

## Data Availability

The processed data are available from the corresponding author upon reasonable request.
