# Supplementary material for: Artificial-Intelligence-Driven Electromyography Adaptation for Elderly Assistance at Physiological, Functional, and Behavioral Levels
Source: Cyborg Bionic Syst. 2026 Jul 15;7:0638. doi: 10.34133/cbsystems.0638 (PMC13369311; doi:10.34133/cbsystems.0638)
Supplement: Supplementary 1 — Supplementary Text Figs. S1 to S3 Movies S1 to S3 [file cbsystems.0638.f1.zip › Supplementary Materials_260428.docx]

Supplementary Material

AI-Driven EMG Adaptation for Elderly Assistance at Physiological, Functional, and Behavioral Levels

Jiaqi Xue, Ziqi Li, Xiaoyang Zou, Zijia Qu, Shengjie Yang, Colin Pak Yu Chan, Yanchen Liu, Zhou Zhao, Jing Zhang, Clio Yuen Man Cheng, Haiyang Wang, Kehan Zou, Yafei Zhao,

Vivian Weiqun Lou, Ning Xi, King Wai Chiu Lai

**Model Comparison**

To further evaluate the capability of the proposed model, we compared it with two representative baseline models that are also suitable for online EMG analysis. The first model is a classic machine learning approach based on hand-crafted EMG features and a random forest classifier. For each EMG window, we extracted three commonly used features: mean absolute value (MAV), root mean square (RMS), and waveform length (WL), and used them as input to the random forest model. The second baseline model is a temporal deep learning model based on a long short-term memory (LSTM) network. This model consists of an LSTM layer, a fully connected layer, and two output layers, and directly uses the raw EMG signal within each window as input. For a fair comparison, all three models were trained and evaluated using the same window length, batch size, data split, and training protocol as our proposed backbone model. In addition, five-fold cross-validation was performed for all three models to provide a more robust evaluation of model performance.

We compared the accuracy and F1-score among models, as shown in Fig. S1 (A)(B). The central line indicates the mean, and the whiskers represent the minimum and maximum values, with individual subject data points overlaid. Among the three models, the proposed backbone model achieved the highest classification accuracy and F1-score, slightly outperforming the LSTM baseline model. The performance gap between the backbone model and the LSTM model was relatively small, indicating that both models could effectively capture discriminative temporal patterns from EMG signals. In contrast, the performance of the random forest model was significantly lower than that of the two deep-learning-based models, suggesting that the hand-crafted features are insufficient to capture discriminative temporal patterns in EMG signals.

We also compared the training and inference efficiency of these models on an NVIDIA RTX 6000 Ada GPU (48 GB memory). Although the LSTM model achieves similar decoding performance to our backbone model, its training time is significantly longer and its inference efficiency is lower, as shown in Fig. S1 (C). This higher computational cost may limit its practicality in real-time EMG decoding and control scenarios. Random forest is excluded from the training-time plot due to its non-epoch-based training. It is the fastest, with 1-fold training time ranging from 8 to 37s. Although the random forest model is computationally efficient, its lower accuracy makes it less suitable for reliable online control. In conclusion, these results demonstrate that our proposed backbone model achieves an optimal balance between decoding accuracy and computational efficiency, making it suitable for real-time EMG analysis and assistive control systems.


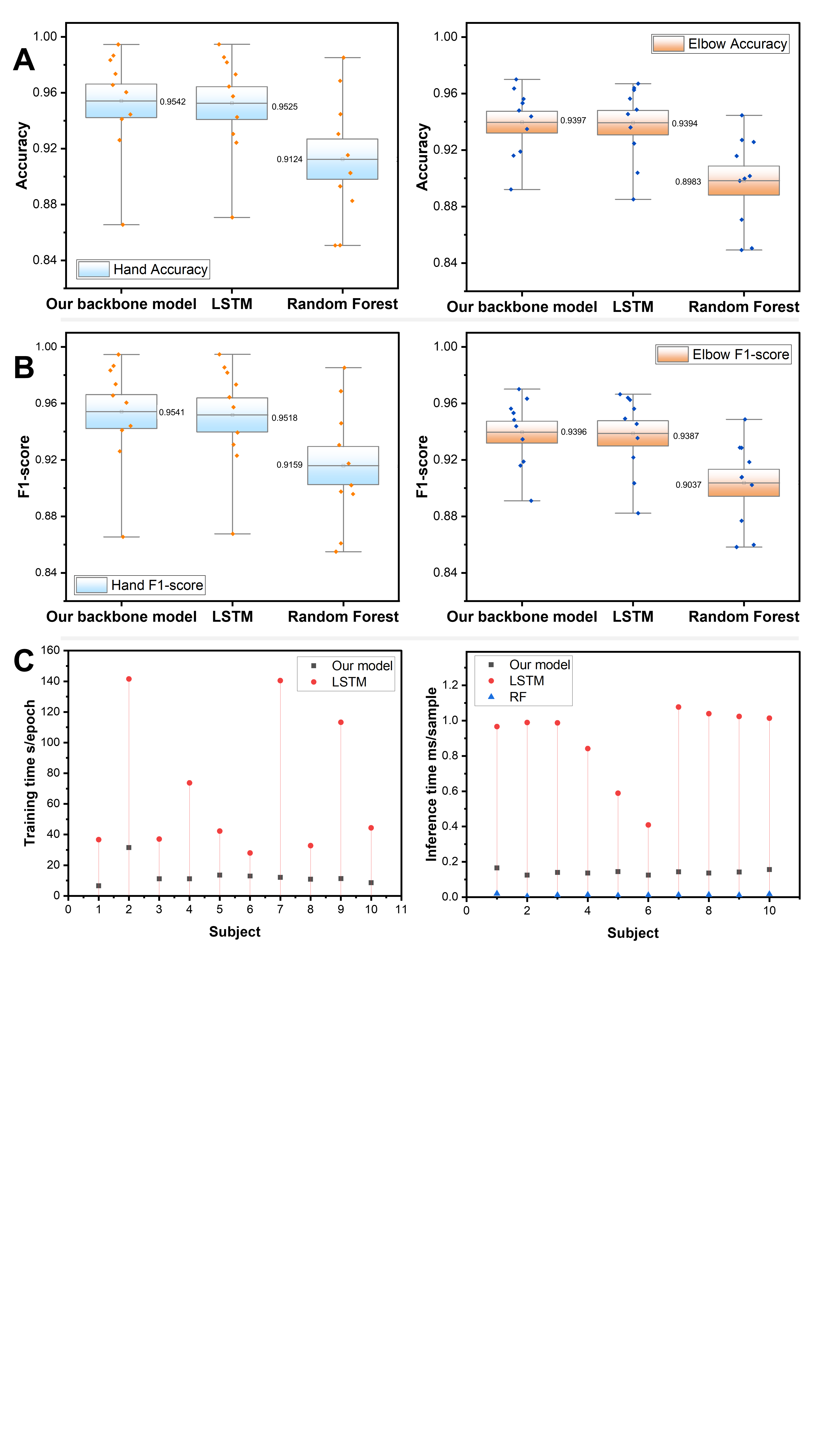


***Fig. S1.*** *Model comparison among our backbone model, LSTM, and random forest. (A) Comparison of prediction accuracy. (B) Comparison of prediction F1-score. (C) Comparison of training and inference time.*

**Additional ADL Task Adaption**

To further evaluate the applicability of the proposed three-level framework, we also evaluated two ADL-related tasks: carrying a bag and pulling a grocery cart. Compared to cooking, these tasks involve different coordination patterns between hand and elbow movement, thus enabling a more comprehensive assessment.

**Pulling a grocery cart**. The prediction curves for this task are shown in Fig. S2. This task requires sustained handle grasping together with force-modulated elbow control during uphill pulling. To compensate for the missing elbow-extension information, a small amount of corresponding data was included in the target data. The distilled model still captures the main behavioral stages of the movement, including hand opening, grasping the cart handle, and pulling the cart uphill. Overall, the predicted hand and elbow results remain consistent with the task. Some fluctuations appear after the cart reaches the top of the slope, which is likely associated with the abrupt change in force demand at that stage. For the hand, the original performance was already close to its limit, and it remained basically unchanged after distillation. For the elbow, distillation yields a substantial improvement, increasing accuracy from 0.8399 to 0.9023, while its functional-level performance also remained at a high level of 91.98%.


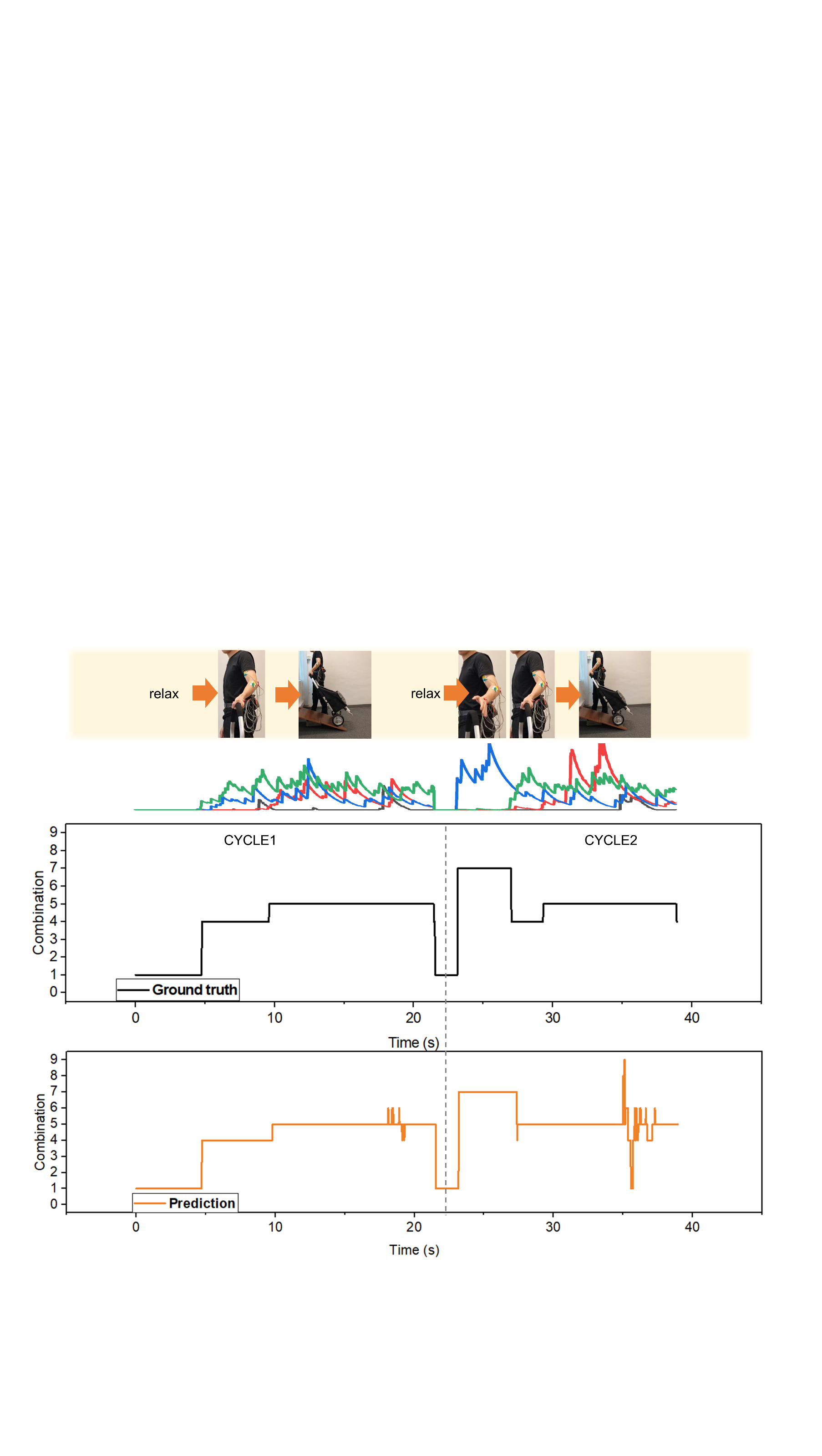


***Fig. S2.*** *Evaluation performance of lifting a bag via knowledge distillation.*

**Lifting a bag.** The prediction curves are shown in Fig. S3. We used only a small amount of data from lifting a bag to distill the model. The distilled model captures the major behavioral phases of the task in a temporally coherent manner, including hand opening, grasp formation, upward lifting, downward lowering, and hand release. Across the sequence, the predicted hand and elbow results remain well aligned with the task progression, with only minor transient fluctuations. Quantitatively, distillation improves both behavioral performance, with the hand accuracy increasing from 83.36% to 87.64% and the elbow accuracy increasing from 84.63% to 89.96%. At the same time, the accuracy on the original functional-level performance decreases by only 0.45%–1.6%. These results indicate that the proposed distillation enhances behavior-level decoding for both hand and elbow while largely preserving the underlying functional intent.


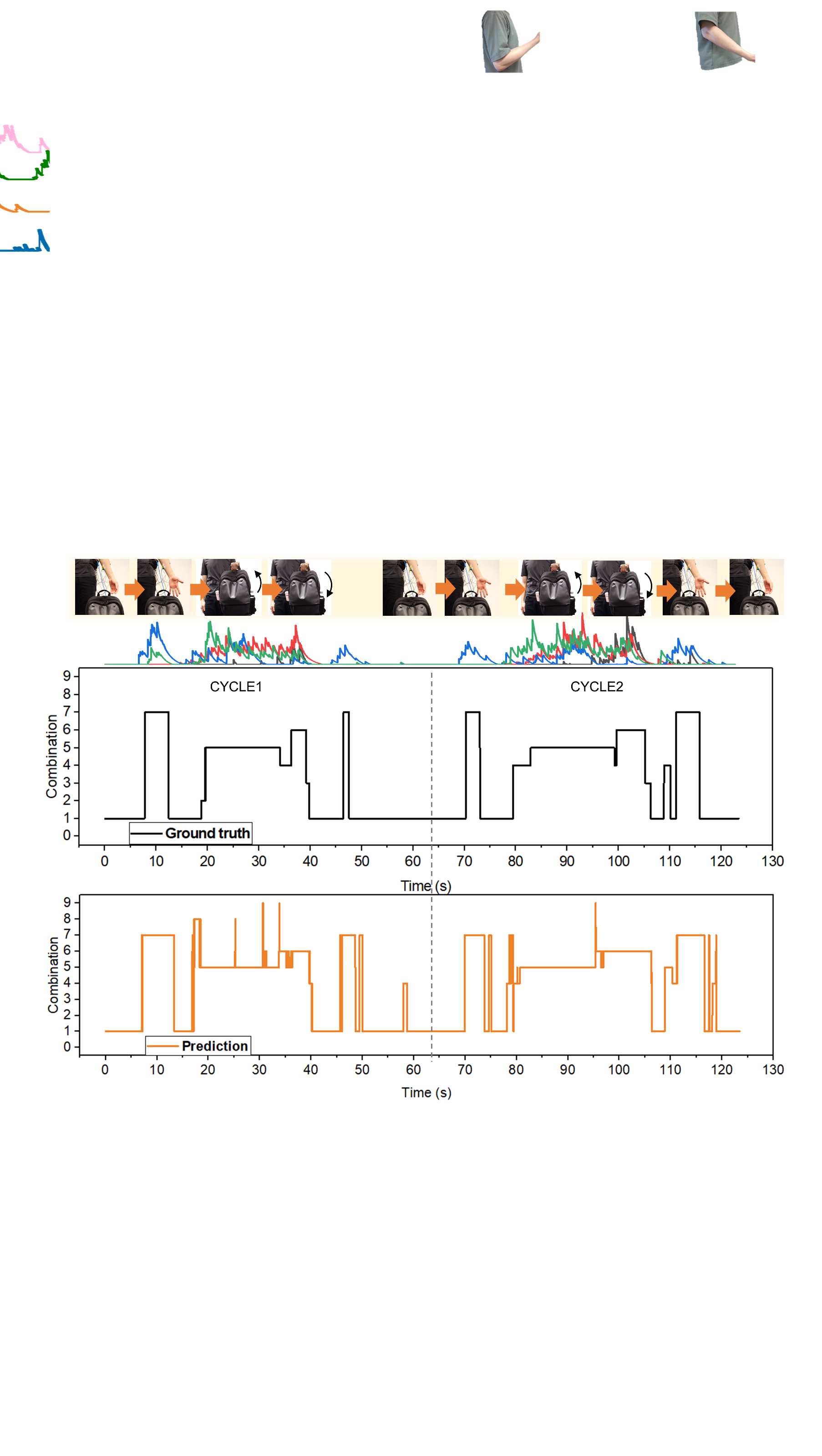


***Fig. S3.*** *Evaluation performance of lifting a bag via knowledge distillation.*

Overall, these additional experiments provide further evidence that the proposed framework can preserve functional intent while producing stable and expressive behavioral-level predictions across a broader set of ADL-related scenarios.
