# Supplementary figures and images for: Artificial-Intelligence-Driven Electromyography Adaptation for Elderly Assistance at Physiological, Functional, and Behavioral Levels
Source: Cyborg Bionic Syst. 2026 Jul 15;7:0638. doi: 10.34133/cbsystems.0638 (PMC13369311; doi:10.34133/cbsystems.0638)

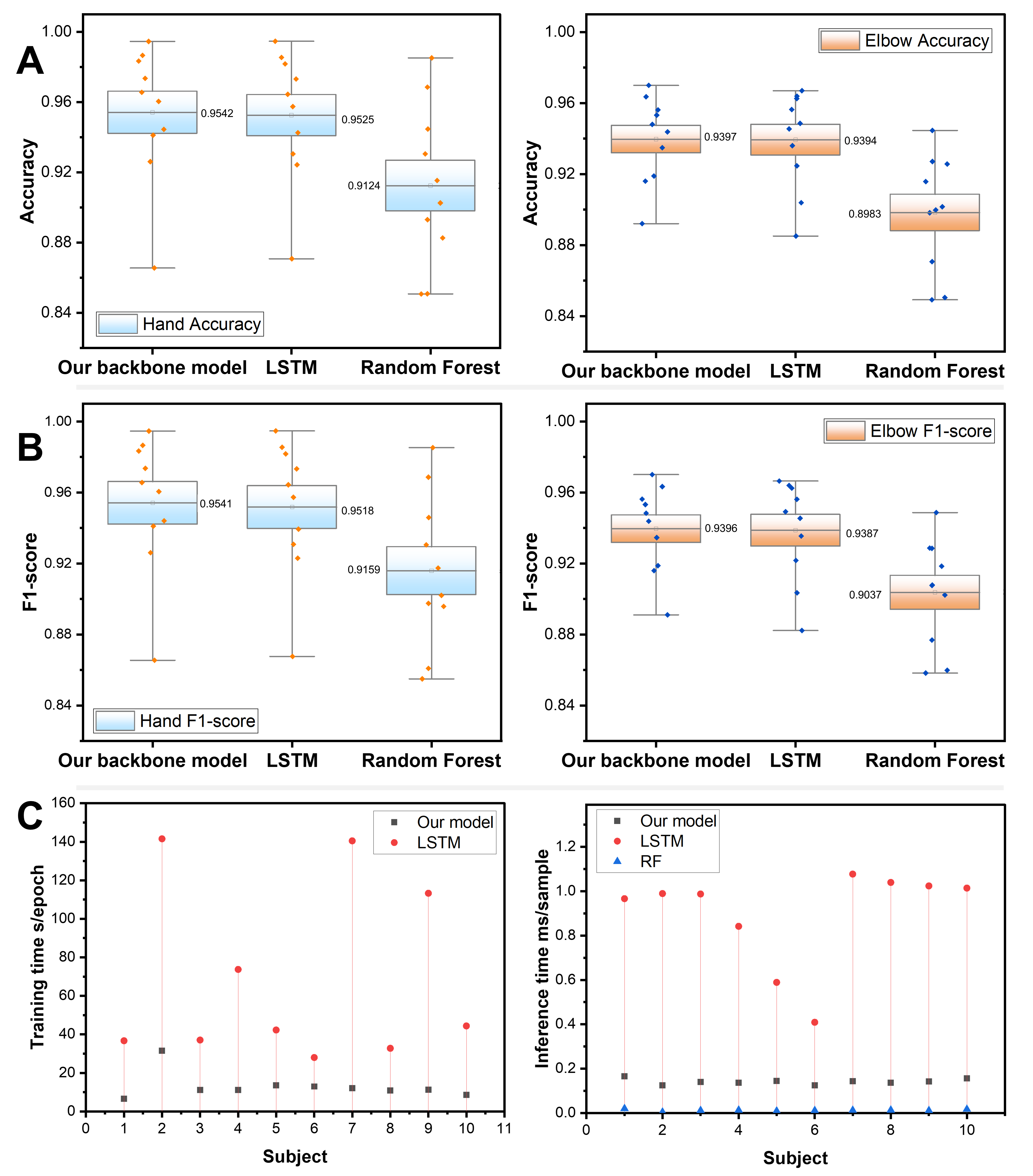

Supplement: Supplementary 1 — Supplementary Text Figs. S1 to S3 Movies S1 to S3 [file cbsystems.0638.f1.zip › figS1.png]

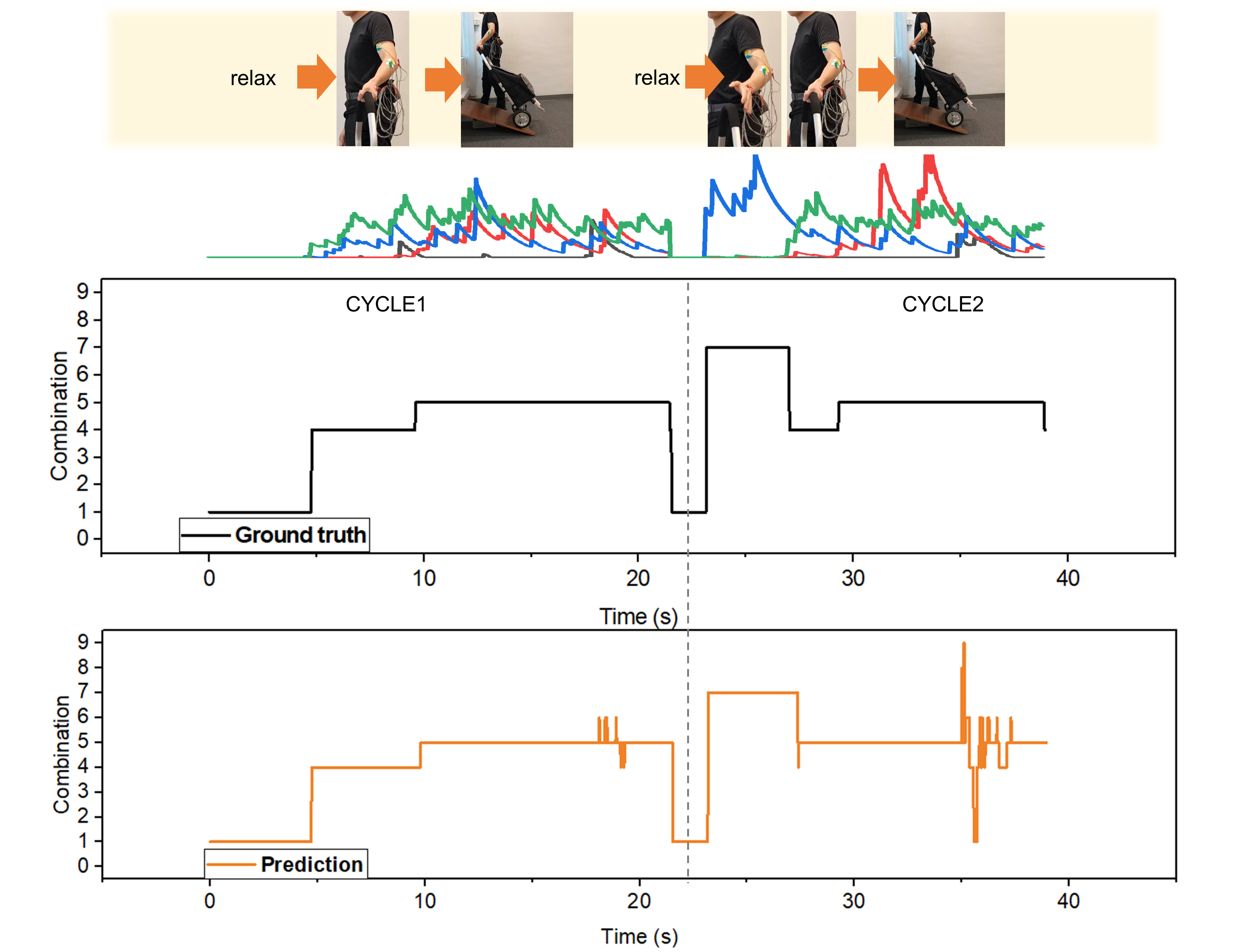

Supplement: Supplementary 1 — Supplementary Text Figs. S1 to S3 Movies S1 to S3 [file cbsystems.0638.f1.zip › figS2.png]

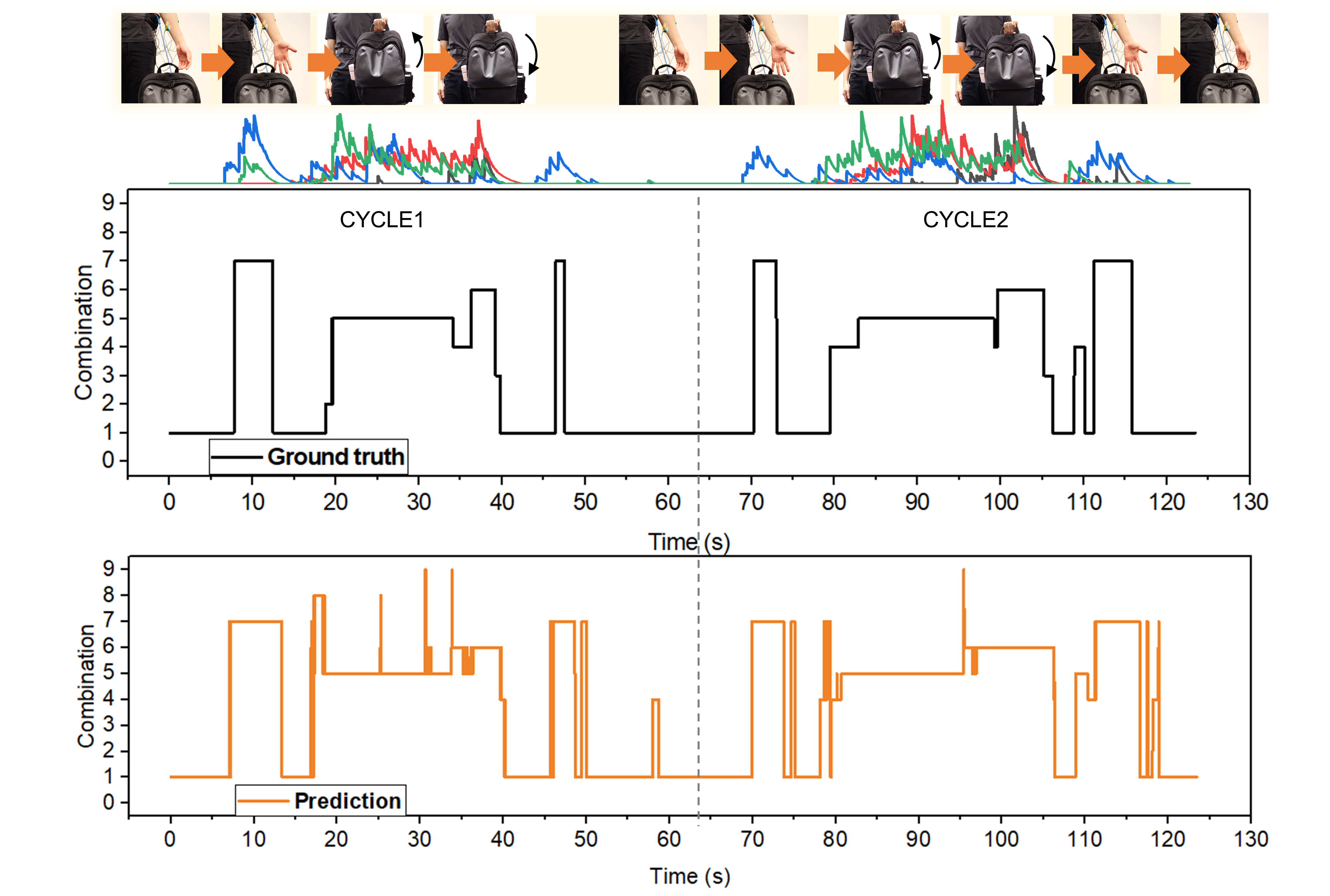

Supplement: Supplementary 1 — Supplementary Text Figs. S1 to S3 Movies S1 to S3 [file cbsystems.0638.f1.zip › figS3.png]
